# Delirium in the Acute Care Setting From the Families Perspective: A Scoping Review

**DOI:** 10.1111/jan.16891

**Published:** 2025-03-30

**Authors:** Amber Mclean, Beverley Ewens, Amanda Towell‐Barnard

**Affiliations:** ^1^ Edith Cowan University Perth Western Australia Australia; ^2^ School of Nursing and Midwifery, Edith Cowan University Perth Western Australia Australia

**Keywords:** acute care, delirium, families experience, family centred care, nursing

## Abstract

**Aim:**

To explore the existing literature on delirium within the acute care setting from the family members' perspective and summarise key findings.

**Design:**

A scoping review guided by Arksey and O'Malley's methodological framework and refined by the Joanna Briggs Institute.

**Review Methods:**

The Population, Concept, and Context framework recommended by the Joanna Briggs Institute's scoping review protocol identified the main concepts in the primary review question. The inclusion criteria focused on primary research studies from any chronological date that explored the family members' experience of delirium within the acute care setting. Following screening by two independent reviewers, data extraction was conducted and presented in tabular form, detailing the study aim, sample, setting, methods, key findings and recommendations for future research and clinical practice.

**Data Sources:**

A comprehensive search was conducted in January 2025 using CINAHL+, MEDLINE, JBI, Cochrane Library, Web of Science, Scopus and Google Scholar. Citation searching and reference lists supplemented this review to identify relevant studies.

**Results:**

Seventeen studies met the inclusion criteria. Families' experiences of delirium were categorised into (1) lack of awareness and understanding of delirium; (2) communication and informational needs of family members regarding delirium; (3) the emotional impact delirium has on family members, and (4) family desire to participate in their loved one's care.

**Conclusion:**

This review highlighted a paucity of literature addressing the experiences of family members who witness delirium in the acute care setting. The existing research underscored the need for clear communication and information regarding delirium to mitigate the negative emotional impact that delirium places on families.

**Impact:**

This scoping review provides insights into the challenges facing families witnessing delirium in the acute care setting. A better understanding of family members' experiences can guide the development of a supported family‐centred approach to delirium care.

**Patient Contribution:**

No patient/public contribution.

## 1 Introduction

Delirium is described as a temporary condition that is characterised by an abrupt change in the brain that causes mental confusion and emotional disruption, which makes it difficult to think, remember, sleep and concentrate (Mart et al. 2021). It represents a significant health concern, affecting approximately 23% of older adults admitted into general medical settings, with notably higher rates of delirium seen in critical care areas and within palliative care (Wilson et al. 2020). Therefore, this scoping review addresses families' perceptions and experiences of delirium within the general acute care setting. The acute care setting encompasses a range of healthcare environments, including emergency medicine, trauma care, ICU, acute care surgery, critical care, urgent care and short‐term inpatient stabilisation, where there is a risk of delirium developing (Department of Health Victoria 2023). The consequences of delirium are profound and can have a long‐lasting impact on patients, families and health organisations (Petrinec and Martin 2018). Delirium has been associated with increased patient mortality and morbidity rates, long‐term cognitive dysfunction, prolonged hospital length of stay, an increased risk of falls and a diminished functional status post‐delirium (Salluh et al. 2015; Klein Klouwenberg et al. 2014; Kotfis et al. 2018; Witlox et al. 2010). Additionally, delirium contributes to increased healthcare costs due to extended hospital stays, increased staffing levels and adverse patient outcomes, placing financial strain on healthcare organisations (Gou et al. 2021).

For families, witnessing the distressing symptoms associated with delirium can lead to heightened stress, anxiety and depression, with some family members developing post‐traumatic stress disorder (PTSD) (Grover and Shah 2013; Kim et al. 2022). As delirium has an impact on everyone involved, including the person with delirium, healthcare organisations and family members, it is of international relevance that delirium is explored from all angles, including from the family members' perspective, to allow for a better understanding of their needs, leading to improved patient outcomes.

## 2 Background

Traditionally, pharmacological interventions including the use of antipsychotic drugs were used as the primary management for delirium and are still commonly prescribed in clinical practice. However, these drugs are no longer recommended due to their inability to treat the underlying cause and their potential side effects which can cause serious harm (Mart et al. 2021). Current delirium management approaches should focus more on nonpharmacological interventions including the active involvement of family members in patients' recovery (National Institute for Health and Clinical Excellence [NICE] 2019). The term family in this review encompasses the modern definition of ‘family by choice’ where anyone deemed significant to the patient, including blood relatives, friends and neighbours (Hull and Ortyl 2019). Family‐centred care (FCC) is becoming increasingly popular within the acute care setting, aiming to respect patients' beliefs, values, preferences, collaboration and family members' participation in patient care (Lange et al. 2022b; Hamilton et al. 2020; Davidson et al. 2017). This approach is exemplified in the ABCDE (Assess pain, Breathing spontaneously, Choice of sedation, Delirium screening and management, and Early mobilisation) multi‐component approach to delirium prevention and management in intensive care units (ICUs) which has now added F for family engagement and empowerment to their holistic approach to delirium management (Pun et al. 2019). Family members, through their in‐depth knowledge of the patient, can provide a sense of security in an unfamiliar environment as well as act as an advocates to ensure the patient's preferences are recognised and respected (Pun et al. 2019; Eghbali‐Babadi et al. 2017; Paulson et al. 2016). However, family involvement can strain the emotional health and well‐being of the family members themselves, underscoring the need to understand their experiences in the clinical setting, which to date has been limited in research.

While family presence has clear benefits for the patients, the psychological consequences for the family members present are well reported. Post‐Intensive Care Syndrome in Family Members (PICS‐F), recognised by the Society of Critical Care Medicine in 2012, describes the psychological symptoms experienced by family members of ICU patients, including depression, anxiety and trauma‐related conditions such as acute stress disorder and PTSD (Serrano et al. 2019). PICS‐F has been recognised as a concerning issue, with the prevalence estimated to be as high as 20%–40% in family members whose loved ones have been admitted to a general ICU (Petrinec and Martin 2018; Davidson et al. 2012; Jones et al. 2004). The recognition of PICS‐F highlights the crucial role of family members in the recovery process of their loved ones but also acknowledges the stress and challenges they face during this process (Petrinec and Martin 2018; Assa et al. 2021). These challenges of experiencing a serious illness in a loved one, coupled with the unpredictability and uncertainty of delirium, make family members at increased risk of developing PICS‐F (Davidson et al. 2012; Petrinec and Martin 2018). The symptoms of PICS‐F can impact both the patient's and family member's recovery and quality of life, highlighting the family members' needs and experiences within the acute care setting beyond the ICU (Petrinec and Martin 2018; Assa et al. 2021).

Existing research predominately focuses on the benefits of family involvement on patients' outcomes as well as presenting ways to achieve more active family involvement in delirium care within an intensive care setting (Pun et al. 2019; Eghbali‐Babadi et al. 2017; Paulson et al. 2016). However, within the current body of evidence, there is a limited insight into the family members' own experiences of delirium in a loved one. While many studies have explored the families' experiences of delirium within the ICU or palliative care settings, delirium impacts people across various acute care settings, necessitating the need for further exploration of family involvement across this population. This scoping review methodology is well suited for this review as it allows the exploration of broader questions to provide an inclusive overview to map the existing evidence in this area (Munn et al. 2022).

This review aims to explore family members' experiences, attitudes and perceptions of delirium in a loved one within the acute care setting, providing a holistic perspective on the existing literature. This review also sought to identify gaps in the existing literature for future research, which could contribute to the development of a more FCC approach to delirium care in the future. By exploring the experiences of family members who witnessed delirium in a loved one, healthcare professionals can gain insights into the challenges family members face and the support they require during this time. Ultimately, this insight could inform the development of support systems for families, information and education programmes for family members about delirium, and improve collaboration between healthcare professionals and families, with the goal of improving the well‐being of both patients and their families, promoting a more FCC approach.

## 3 The Review

### 3.1 Aim

This scoping review aimed to explore the existing literature on delirium within an acute care setting from the family members' perspective.

### 3.2 Design

This scoping review was conducted following the methodological framework developed by Arksey and O'Malley (2005), further refined using guidance from the Joanna Briggs Institute (JBI) (Peters et al. 2020). The review also adhered to the PRISMA‐ScR scoping review protocol (Preferred Reporting Items for Systematic Reviews and Meta‐Analyses) (Tricco et al. 2018). The question that guided this scoping review was ‘What are the family members' experiences of delirium within the acute care setting?’

### 3.3 Search Methods

The search strategy aimed to locate relevant published and unpublished studies, following the three‐step process recommended in the JBI evidence synthesis (Peters et al. 2020). In step one, a limited search was conducted in MEDLINE and CINAHL+ to compile a list of key terms and Mesh terms. This step also served as an opportunity to identify any current scoping reviews in this area. Table 1 illustrates the keywords from the titles and abstracts of relevant articles and index terms that were used to formulate a full comprehensive search strategy (Appendix S1 provides the full keyword search details). The most recent searches were conducted between April 2024 and January 2025 to ensure the inclusion of the most up‐to‐date studies. In step two, online databases including CINAHL+, MEDLINE, JBI, Scopus, Web of Science, Cochrane Library and Google Scholar were searched, and relevant studies were identified. Grey literature including the World Health Organization (WHO) website and the Australian delirium standards was also searched for relevant information and data. Step three involved screening the reference list of relevant studies and including them if they met the inclusion criteria for the scoping review.

**TABLE 1:** Search terms of logic grid.

Population	Concept	Context
Family members	Experiences of delirium	In acute care settings
Family	Delirium	Hospital
Carer	Psychosis	Intensive care units
Relatives	Experience	Acute care
Loved ones	Delirium	Post operative
Caregivers	Families Perspective	MH‐ Acute care
MH‐ Extended family	Families Attitudes	
MH‐Family	Delirium prevention	
	Families View	
	MH‐ ICU psychosis	
	MH‐ Delirium	

### 3.4 Inclusion Criteria

Primary research studies including quantitative, qualitative and mixed methods study designs, in the English language were considered for this review. To ensure all relevant studies were identified, the reference list of the review paper was searched. As recommended by the Johanna Briggs Institute (JBI) methods, inclusion criteria were based on the Participants, Concept, and Context (PCC) described in the following sections (Peters et al. 2020).

### 3.5 Participants (P)

Participants in this scoping review were family members of individuals diagnosed with delirium within the acute care setting. As previously stated for this review the definition of a family member is anyone deemed significant to the person with delirium, adopting the concept of ‘family by choice’, including partners, children, siblings, relatives, friends, or neighbours (Hull and Ortyl 2019). Only studies whose participants were over the age of 18 years old were included, due to concerns of co‐founding factors outside the scope of this review.

### 3.6 Concept (C)

The concept for this scoping review is the experiences, attitudes and perspectives of family members regarding delirium. The focus is specifically on the experiences of families in relation to delirium in their loved ones, rather than any delirium management interventions that involved family members.

### 3.7 Context (C)

The context for this review was the acute care setting, which encompasses a range of healthcare environments, including emergency medicine, trauma care, ICU, acute care surgery, critical care, urgent care and short‐term inpatient stabilisation (Department of Health Victoria 2023). This review does not address studies conducted within palliative care, community care, or aged care primarily due to concerns related to transferability.

### 3.8 Study Selection

**FIGURE 1:**
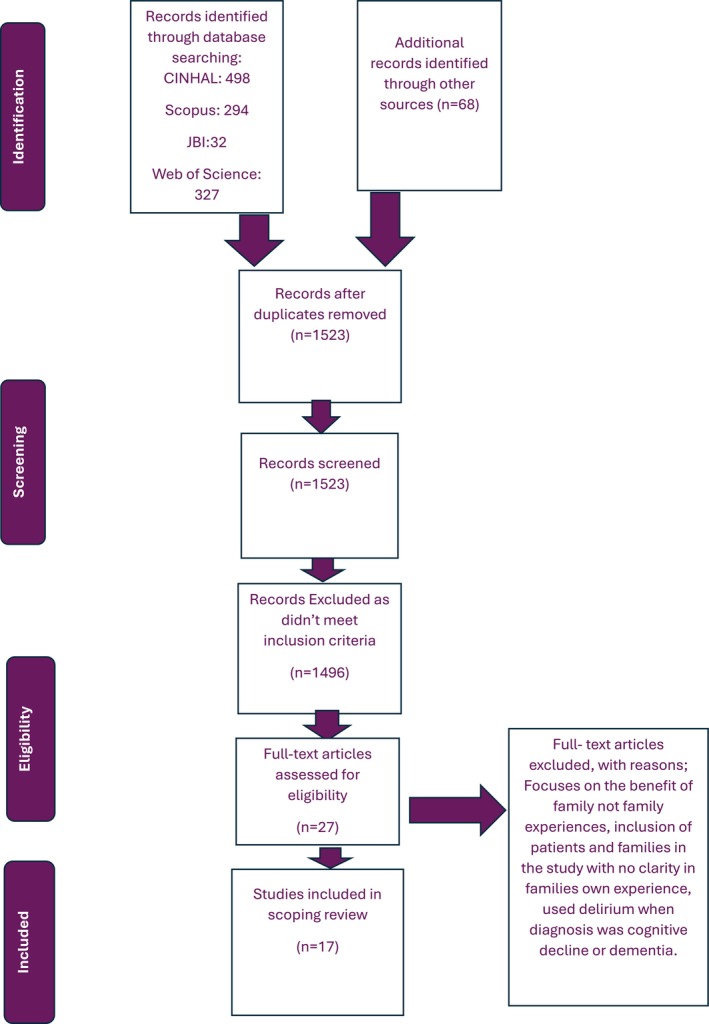
PRISMA‐ScR flow diagram (Tricco et al. 2018).

Following an extensive search, all identified citations were uploaded onto EndNote Version 20 (Clarivate Analytics 2018). After duplicates were removed, titles were screened against the inclusion criteria. Abstracts were then screened, and relevant studies were uploaded to the software package Rayyan (Ouzzani et al. 2016), with the blind mode on to limit reviewer bias and record screening decisions and justifications. Full‐text studies were independently reviewed by two reviewers to assess eligibility, with a third reviewer consulted to resolve any disagreements. The search results and the study inclusion process are presented in a Preferred Reporting Items for Systematic Reviews and Meta‐analyses extension for scoping review (PRISMA‐ScR) flow diagram included in Figure 1 (Tricco et al. 2018).

### 3.9 Search Outcomes

The initial database search yielded a total of 2816 results with 1523 duplicates identified and removed. Twenty‐seven studies were considered for full‐text eligibility, and seventeen were ultimately included in the review following the two‐person independent review process. Studies were excluded when the study did not exclusively focus on the family's experience of delirium. Additional reasons for exclusion included two studies that focused on the development of a nursing intervention rather than the families' experiences, and one study focused on the benefits of early mobilisation on the reduction of delirium and families' perceptions of that specific intervention. Two studies were literature reviews and not primary research articles therefore their reference list was reviewed for any studies that met the inclusion criteria of the review. Two initially selected studies were later excluded as upon close examination they were conducted outside the acute care setting with one within a community day centre, and the other within an advanced cancer ward. The other three studies were excluded due to their focus on the importance of the family's role in relation to the patient's overall experience rather than the families' experience.

### 3.10 Quality Appraisal

In line with the scoping review methodology, critical appraisal of the included studies was not conducted, as the objective of this review was to describe and map the existing body of literature rather than assess study quality or risk of bias (Pollock et al. 2021).

### 3.11 Data Charting and Extraction

A data extraction table adapted from the JBI scoping review method (Peters et al. 2020) was used to systematically collect relevant information. The extracted data is organised and shown in Table 2, and includes the authors, publication date, study aim, methodology, context, data collection and analysis methods, findings and areas for future research and clinical implications.

**TABLE 2:** Extracted data.

Author	Aim	Design	Sample	Data collection/analysis	Findings	Future research/clinical recommendations
Bohart et al. (2019)	To explore family members experience of delirium in critically ill patients admitted to ICU	Qualitative phenomenological approach	11 family members of critically ill patients admitted to a general medical/surgical level 2 ICU in Denmark	Semi‐structured interviews. Recorded and used NVivo	Delirium was not the main concern due to a lack of understanding. Resulting in family members making up their own reasons for the confusion. Communication with healthcare professionals is crucial. Uncertainty about patients' future was a concern for family members	Investigate the potential ways to assist family members to develop coping strategies to help deal with delirium. Family member's need for information was highlighted and could be further investigated
Cohen et al. (2019)	To understand how family members of older adults hospitalised for orthopaedic surgery are integrated by nurses in delirium prevention	Qualitative multiple case study approach	8 case studies composed of one nurse, one patient and one family caregiver in French Switzerland	Semi‐structured interviews and family member logs	Most family caregivers wanted to be involved in care despite challenges. Limited recognition of family members' role by nurses. Barriers to communication included institutional and organisational procedures. Family members experienced stress, frustration and confusion due to the lack of integration of family members in the care team	An open culture to include family members in patient care should be welcomed and explored how to better achieve this. The study was limited due to gender bias majority of participants were female a larger sample could be studied
Day and Higgins (2015)	To explore the experiences of family members during their older loved one's delirium	Qualitative phenomenological study	14 family members across multiple care settings in Australia	In‐depth interviews. Thematic analysis	Sudden unexpected absence of family members during delirium leads to distress and shock. Description of ‘living with a stranger’ and Life on hold ‘waiting for loved one to return’ highlighting the uncertainty of delirium. ‘In the dark’ due to poor communication and education. Being ‘pushed aside’ by healthcare staff, on the fridge, not centre stage highlights the lack of support and communication from healthcare staff	The importance to acknowledge the family's experience with delirium was highlighted and should be transferred into clinical practice. The study included delirium on dementia but focused on delirium and highlighted the difference between delirium and dementia with the sudden nature of delirium. This could be further explored
Grover and Shah (2013)	To study family members' distress with symptoms of delirium	Quantitative prospective study design	53 patients and their 72 family members were included in a hospital in India	Questionnaire developed for this study. Data analysed using SPSS‐14	Delirium causes significant distress to the family members. Distress increased with more symptoms of delirium present particularly hyperactive delirium	Further development of the questionnaire with the input of nurses who are involved in the day‐to‐day assessment of patients with delirium. Highlighted the importance of giving family members education on the short‐lasting nature of delirium
Huang et al. (2022)	To understand the experience of family members who have a family member as a patient with delirium in the ICU in Taiwan	Qualitative descriptive approach	20 family members of patients in ICU in Taiwan	Face‐to‐face semi‐structured interviews. Thematic analysis	The perplexity of the ICU environment‐ restricted visiting made it seem a scary environment. The perplexity of making decisions‐ lack of knowledge of delirium and medical care made it very hard for families to make any care decisions. The perplexity of Chinese cultural constraints	Improved communication is needed to reduce uncertainty. Information access could be enhanced through smartphone QR codes future research is needed
Kim et al. (2022)	To identify depressed moods and associated factors in family members of patients with delirium in general wards and explore family members' knowledge and understanding of delirium and nonpharmacological management	Quantitative cross‐sectional survey design	224 family members participated. Based in a Korean psychiatry department within the hospital	Statistical analysis	Family misunderstood delirium, only 13% correctly identified delirium. 74% received no information about nonpharmacological management of delirium. 54% reported depressed mood	Further research into family members' knowledge of nonpharmacological logical management. Differences between hepatic encephalopathy and delirium may have confused participants
Lange et al. (2022a)	To explore patient and families experience of delirium during their stay in ICU	Qualitative phenomenological study	8 participants pairs (family and patient) from a cardiac ICU in a teaching Hospital in Poland	Semi‐structured interviews one month after discharge. Thematic analysis	Education‐ lack of education specifically before admission to the hospital. Feelings of distress identified. Family members struggled to discuss delirious episodes with the patient afterward to avoid distress and embarrassment	Larger sample size needed to fully assess experiences. Importance of education highlighted
Martins et al. (2018)	To analyse the distress levels caused by a delirious episode in patients, families and nurses	A quantitative prospective pilot study	42 inpatients, 32 family members and 12 nurses from two general medical ICUs in a university hospital in Portugal	Data collection complied from 7 different scoring systems	The family experienced more distress than the nurses or the patient. Distress over symptoms of excessive drowsiness and agitation	Further research into education and psychological support
Meilak et al. (2019)	To describe the experiences of post operative delirium and explore the views of patients and family members to inform the co‐design of an intervention to minimise distress related to post operative delirium	Qualitative study	11 patients and 12 family members in a London hospital	Semi‐structured interviews. Recorded transcribed then thematic analysis done. NVivo used	15/23 had not heard of delirium prior, as a result, the family felt frustration, anger and worry. Family members felt distress due to observing the delirium. Lack of knowledge family came up with their own ideas of causes of confusion. Good communication was highlighted as essential	Staff training and enhanced public awareness of delirium. Further research into the effectiveness of the intervention was developed regarding information and support needs of family members
Pandhal and Van Der Wardt (2022)	To explore the perception of ICU patients and their families in relation to the involvement of family in delirium management	Qualitative exploratory study	18 participants‐ 9 patients and 9 family members from a UK hospital	Semi‐structured interviews of ICU patients and their family members together. Thematic analysis is done	Participants lacked an understanding of delirium. Influencers of delirium management families and healthcare professionals‐ the importance of good communication. Family‐based delirium care is important to reduce anxiety, but more education and information are required from healthcare staff	Further research is to investigate strategies and interventions to understand family members influence on delirium management in ICU
Poulin et al. (2021)	Examine the association of patient delirium in ICU with patterns of anxiety symptoms in family members	Quantitative cross‐sectional survey	147 family members of patients admitted to ICU in a Canadian hospital	Through data analysis of self‐reported anxiety level and CAM ICU and sour 7 assessment	44% of family members reported feelings of anxious or on edge. 44% of family members reported not being able to stop or control worrying or worrying too much about different things	Development of mental health interventions for the diversity of anxiety symptoms experienced by family members of critically ill patients are needed. Longitudinal studies are needed to develop treatments and interventions for family anxiety
Rosgen et al. (2021)	To evaluate associations between family members detected delirium in critically ill patients and depression and anxiety symptoms in their family members	Quantitative cross‐sectional study	147 patient and family members pairs. Based in a 28 bedded medical/surgical ICU in Canada	Questionnaire using self‐reported symptoms for anxiety and depression symptoms. Descriptive statistics and regression analyses were completed	Anxiety and depression symptoms in 27% and 35% of family members respectively. No association between delirium detected by family members using assessment tools and anxiety and depressive symptoms	Further randomised research is required to confirm these associations
Russ et al. (2019)	To advance understanding of the family members and patients with delirium and demonstrate how experience‐based design supported co design of novel tools to aid patients and family members in identifying delirium and mitigating the consequences	Mixed methods experience‐based design	Interviews from 10 patients, 4 family members and 16 staff members across critical care and acute care settings. Questionnaires to 31 patients, 38 caregivers and 61 staff members. Pacific Norwest USA Hospital	Observations, interviews and questionnaires were used. The tools and processes were developed to enhance family members education about delirium. Thematic analysis through the grounded theory approach	The family felt underprepared regarding delirium‐ lack of knowledge led to fear, anxiety, distress, anger and uncertainty. Family members felt communication between staff could be improved. Lack of education preadmission noted (95%). Family members felt overwhelmed and underprepared once the patient was discharged	Larger samples are needed for true representation of data
Schmitt et al. (2019)	To describe the common burdens of patients, family members and nurses	Qualitative interview study	18 patients, 16 family members and 15 nurses participated. Based in a large urban hospital in Boston, USA	Semi‐structured individual interviews. Thematic analysis is done	Family members struggled when patients were unable to recognise them causing uncertainty. Symptom burden of hallucination, disorientation and changes in personality were burdensome. Emotional burden of feelings of anger, helplessness and distress was noted. The situational burden of loss of control, lack of support and lack of knowledge were identified	Importance of prevention tools and programmes highlighted to prevent this emotional and stressful burden of delirium for all people involved in the delirious episodes. Further research into enhancing person and family centred care approach in delirium care
Smithburger et al. (2017)	To gain insight into the opinions of patient's families regarding active participation in delirium prevention activities to inform specific recommendations for involving patients' families in such activities	Qualitative grounded theory	10 family members from a 24 bedded ICU in the USA	Semi‐structured interviews. Thematic analysis is done	Consistent family presence and participation of care were noted positively with family members expressing a strong desire to be involved in patient care. Family members reported inconsistencies in communication and practices with healthcare staff. The family highlighted that personalised communication and education would be beneficial	Further research into community ICUs and other types of ICUs could identify any disparities between community ICUs and specific ICUs (e.g., cardiac, neurological ICU's)
Stenwall et al. (2007)	To understand the lived experience of family members encountering older persons with acute confusion state	Qualitative descriptive phenomenological study	10 family members in two hospital wards in Sweden	Semi‐structured interviews. Use of Dahlberg analysis	The patient was suddenly unfamiliar due to a sudden change in behaviour. Every encounter is new, and feelings of insecurity, sadness, vulnerability and distress are noted. Experiencing loss. Lack of knowledge and education was noted	Further studies are needed focusing on the experiences of older persons with Acute Confusion Syndrome who encounter family members
Toye et al. (2012)	To describe families' experiences, understandings of delirium and delirium care and support needs	Descriptive mixed methods	12 family members were interviewed from a Delirium‐specific unit in Australia	Questionnaire then semi‐structured interviews. NVivo was used for thematic analysis	Worries, concerns, shock and sadness are felt by families during delirium episodes. Families highlighted the need for communication with healthcare staff. Lack of information led to family members finding their own information about delirium elsewhere. Concerns about discharge and the future were highlighted by family members	Further research into areas outside delirium specific units. Further research into the experiences of male family members should be further explored

### 3.12 Synthesis

Scoping reviews map existing literature and identify areas for future research without making definitive conclusions or clinical recommendations (Pollock et al. 2021). Unlike systematic reviews, scoping reviews focus on summarising evidence descriptively, using methods such as basic coding and frequency counts (Pollock et al. 2021). The main aim of this study was to map the existing literature to identify areas of future research and organise the data on this topic without the need to critically appraise the existing data. For this to occur and to organise and map the data correctly to give a broad overview of family members experiences of delirium within the acute care setting, descriptive qualitative content analysis was undertaken along with frequency concept counts for the quantitative data from the included studies. To prevent repetition, the qualitative and quantitative main findings are presented together as there were key concepts found across all included studies. Combining qualitative and quantitative findings provides a comprehensive understanding of family members experiences with delirium in the acute care setting.

## 4 Results

### 4.1 Characteristics of Studies

#### 4.1.1 Study Design

**FIGURE 2:**
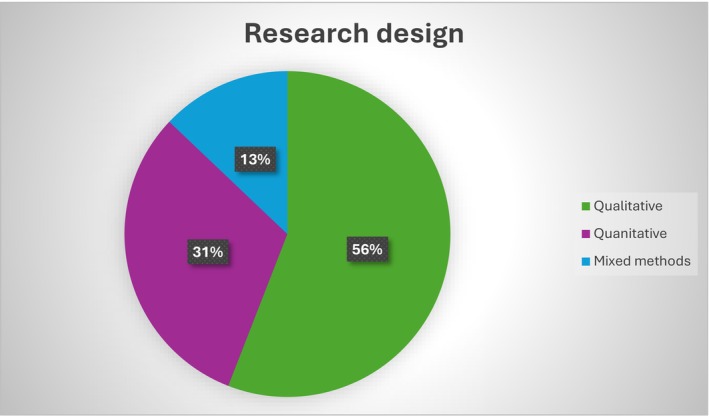
Research design.

Of the included studies, there were 10 qualitative studies (Bohart et al. 2019; Cohen et al. 2019; Day and Higgins 2015; Huang et al. 2022; Lange et al. 2022a; Meilak et al. 2019; Pandhal and Van Der Wardt 2022; Schmitt et al. 2019; Smithburger et al. 2017; Stenwall et al. 2007), with four using a phenomenological approach (Bohart et al. 2019; Day and Higgins 2015; Lange et al. 2022a; Stenwall et al. 2007). Two qualitative studies did not explicitly state their methodology (Meilak et al. 2019; Schmitt et al. 2019). Other qualitative designs included one case study (Cohen et al. 2019), one descriptive study (Huang et al. 2022), one grounded theory study (Smithburger et al. 2017) and one exploratory study (Pandhal and Van Der Wardt 2022). Quantitative studies (*n* = 5) (Grover and Shah 2013; Kim et al. 2022; Martins et al. 2018; Poulin et al. 2021; Rosgen et al. 2021) were represented, with three being cross‐sectional (Kim et al. 2022; Poulin et al. 2021; Rosgen et al. 2021) and two prospective quantitative studies (Grover and Shah 2013; Martins et al. 2018). Additionally, two studies utilised mixed methods approaches (Russ et al. 2019; Toye et al. 2012). Figure 2 illustrates the breakdown of the data research design of the included studies.

#### 4.1.2 Geographical Location

**FIGURE 3:**
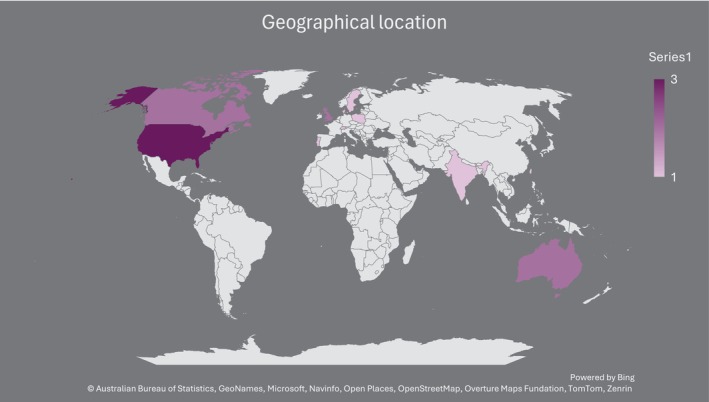
Geographical map of included studies.

The geographic distribution of the studies (*n* = 17) was diverse, providing a wide demographic representation as seen in Figure 3. Three studies were conducted in the United States, with two from Australia, Canada and the United Kingdom. The remaining studies were from Denmark, India, Taiwan, Sweden, Portugal, Korea, Poland and Switzerland.

#### 4.1.3 Focus of the Studies

The primary focus of eight studies was exclusively on family members' experiences within acute care settings (*n* = 8) (Bohart et al. 2019; Day and Higgins 2015; Huang et al. 2022; Kim et al. 2022; Poulin et al. 2021; Smithburger et al. 2017; Stenwall et al. 2007; Toye et al. 2012). In five studies, patients experiencing delirium were also included alongside family members (Grover and Shah 2013; Lange et al. 2022a; Meilak et al. 2019; Pandhal and Van Der Wardt 2022; Rosgen et al. 2021). Four studies examined the experiences of staff, patients and family members concurrently, these studies were included as they provided further insight into the families' experiences of delirium in the acute care setting (Cohen et al. 2019; Martins et al. 2018; Russ et al. 2019; Schmitt et al. 2019). Furthermore, the findings from these studies (Cohen et al. 2019; Martins et al. 2018; Schmitt et al. 2019) aligned with the studies focusing on families' experience alone. All studies focused on the impact delirium had on family members within an acute care setting. Two studies did include family members whose relatives had delirium superimposed on dementia (Day and Higgins 2015; Martins et al. 2018). These were included as the focus was entirely on the families' experiences of delirium and provided insight into the difference between the family members' experiences of delirium compared with their experiences of dementia.

#### 4.1.4 Clinical Setting

Eight of the studies were conducted within the ICU (Bohart et al. 2019; Huang et al. 2022; Lange et al. 2022a; Martins et al. 2018; Pandhal and Van Der Wardt 2022; Poulin et al. 2021; Rosgen et al. 2021; Smithburger et al. 2017) Seven studies were conducted within the wider acute care setting (Day and Higgins 2015; Grover and Shah 2013; Kim et al. 2022; Cohen et al. 2019; Russ et al. 2019; Schmitt et al. 2019; Stenwall et al. 2007). One study focused primarily on postoperative delirium (Meilak et al. 2019) and one study was conducted within a delirium‐specific unit (Toye et al. 2012).

## 5 Main Findings

There were four key concepts identified in both the qualitative and quantitative data, which are presented in Table 3. These include the lack of prior awareness of delirium, the communication and informational needs of family members, the emotional impact that witnessing delirium had on family members and the family's desire to participate in their loved one's care. The integration of qualitative and quantitative data provides a comprehensive understanding of family members' experiences with delirium. While qualitative data offered detailed narratives and in‐depth insights into the emotional and cognitive challenges faced by families, quantitative data quantified the prevalence and severity of these experiences, allowing for the identification of patterns and trends across studies.

**TABLE 3:** Main findings.

Studies	Lack of understanding of delirium	Communication and information needs of the families	Emotional impact delirium had on families	Families desire to be involved in loved one's care
Bohart et al. (2019)	X	X	X	X
Cohen et al. (2019)		X		
Day and Higgins (2015)		X	X	X
Huang et al. (2022)	X		X	X
Lange et al. (2022a)		X	X	X
Meilak et al. (2019)	X	X	X	
Pandhal and Van Der Wardt (2022)	X	X		
Russ et al. (2019)	X	X	X	
Schmitt et al. (2019)	X	X	X	
Smithburger et al. (2017)		X		X
Stenwall et al. (2007)	X	X	X	
Toye et al. (2012)	X	X	X	
Grover and Shah (2013)			X	
Kim et al. (2022)	X	X	X	X
Martins et al. (2018)			X	
Poulin et al. (2021)			X	
Rosgen et al. (2021)			X	

### 5.1 Lack of Understanding and Awareness of Delirium

Both qualitative and quantitative data highlighted a significant lack of awareness and understanding of delirium among family members. Many qualitative studies found that family members lacked prior knowledge, contributing to distress, worry and confusion during delirium episodes (Bohart et al. 2019; Day and Higgins 2015; Huang et al. 2022; Lange et al. 2022a; Meilak et al. 2019; Pandhal and Van Der Wardt 2022; Smithburger et al. 2017; Schmitt et al. 2019; Toye et al. 2012). Similarly, quantitative data revealed that only 13.4% of family members correctly identified delirium, with many misclassifying it as anxiety, dementia, or other conditions (Kim et al. 2022). This lack of awareness hindered their ability to make sense of the situation and to make informed decisions about care (Huang et al. 2022).

Family members often perceived delirium as a natural consequence of critical illness or surgery, leading to unexpected changes in patient behaviour (Bohart et al. 2019; Meilak et al. 2019). Healthcare staff's reluctance to explicitly label it as delirium further contributed to the confusion, as terms like ‘confused’ or ‘not themselves’ were often used instead (Bohart et al. 2019; Day and Higgins 2015; Huang et al. 2022; Lange et al. 2022a; Pandhal and Van Der Wardt 2022; Russ et al. 2019; Toye et al. 2012). Quantitative findings reinforced this, showing the need for improved education to enhance family members' understanding of delirium (Kim et al. 2022).

### 5.2 Communication and Informational Needs

Both qualitative and quantitative data underscored the critical importance of effective communication and information sharing between family members and healthcare staff. Qualitative studies identified inconsistent and inadequate communication between healthcare professionals, relatives and patients as a recurring issue (Lange et al. 2022a; Meilak et al. 2019; Pandhal and Van Der Wardt 2022; Russ et al. 2019; Schmitt et al. 2019; Smithburger et al. 2017; Stenwall et al. 2007; Toye et al. 2012), leaving family members feeling ‘left in the dark’ during delirium episodes (Day and Higgins 2015) This was supported, with 74% of family members reporting that they had never received information on nonpharmacological management for delirium (Kim et al. 2022).

### 5.3 Emotional Impact on Families

The emotional toll of delirium was a consistent theme across both qualitative and quantitative findings. Family members described distress, fear and helplessness while witnessing delirium (Bohart et al. 2019; Day and Higgins 2015). The erratic and unpredictable behaviour of patients often made family members feel ‘like strangers’ and deeply distressed when the patient failed to recognise them (Bohart et al. 2019; Day and Higgins 2015; Meilak et al. 2019). Quantitative data revealed high rates of psychological distress among family members, up to 66.7%, who reported experiencing severe distress when confronted with delirium symptoms in patients (Grover and Shah 2013; Martins et al. 2018). Also, the severity of delirium in patients was found to be consistently linked to increased family members distress, particularly regarding anxiety symptoms (Grover and Shah 2013). These high rates of psychological distress prevalent among family members of patients with delirium included symptoms of depression and anxiety, with 54.9% of family members experiencing depressive symptoms (Kim et al. 2022) and 35.4%–40.4% reporting clinically significant symptoms of anxiety (Russ et al. 2019; Poulin et al. 2021).

Witnessing delirium was described as ‘waiting for a loved one to return’, emphasising the uncertainty family members faced (Day and Higgins 2015). Some family members likened the experience to ‘keeping secrets’ to avoid distressing the patient further, which led to feelings of guilt (Day and Higgins 2015). Family members also respected the patients' wishes to avoid discussing delirium, leaving them to deal with their feelings and concerns themselves (Lange et al. 2022a). The ICU environment and the use of restraints exacerbated these emotions, making family members feel helpless and overwhelmed when they saw their loved ones ‘tied up like prisoners’ (Huang et al. 2022).

### 5.4 Family's Desire to Participate in Care

Family members expressed a strong desire to be more involved in care management but were often excluded or unsure of how they could assist or contribute to their loved one's care (Bohart et al. 2019; Day and Higgins 2015; Smithburger et al. 2017). Family members highlighted a need for personalised guidance to help be involved in care to support patients during delirium episodes and reduce it happening again (Kim et al. 2022). Positive communication and information sharing have been shown to reduce anxiety among family members (Huang et al. 2022). Furthermore, the need for early and personalised information to help families understand and cope with delirium was highlighted (Lange et al. 2022a; Smithburger et al. 2017).

## 6 Discussion

Delirium is a complex medical condition that not only affects patients but also has a profound impact on their families (Lange et al. 2022b). This scoping review systematically maps the existing literature on family members' experiences of witnessing delirium in a loved one within the acute care setting, emphasising their lack of understanding of delirium, their communication and informational needs, the emotional impact of witnessing delirium and their desire to be involved in care.

Family members frequently struggle to understand delirium, its causes, symptoms and management (Toye et al. 2012). Many family members are unfamiliar with the term before their loved one is diagnosed, and some misinterpret delirium as a permanent cognitive decline, such as dementia (Bohart et al. 2019). The unpredictable nature of delirium further compounds this misunderstanding, leaving families uncertain about their loved one's recovery and the best ways to support them (Assa et al. 2021; Day and Higgins 2015). This gap in understanding highlights the need for structured educational initiatives tailored to families' needs, particularly in acute care settings (Smithburger et al. 2017).

The unpredictable symptoms of delirium often cause family members to fear that the changes in their loved one's behaviour could be permanent or indicative of a progressive condition like dementia, adding to their emotional burden (Toye et al. 2012). It is noteworthy that while some family members did not see delirium as a main concern (Bohart et al. 2019) others struggled to grasp the concept of delirium and viewed it as a permanent issue (Toye et al. 2012). This variation in the understanding of delirium further highlights the need for improved information sharing and communication between healthcare professionals and family members, as noted in several of the included studies.

Effective communication between healthcare staff and family members emerged as a critical factor in mitigating distress and improving the overall experience for family members (Smithburger et al. 2017). Studies show that clear and empathetic communication can help family members better understand the nature of delirium, its potential risk factors and the strategies for managing it (Smithburger et al. 2017). However, this scoping review also revealed instances where healthcare professionals did not clearly inform family members about delirium (Bohart et al. 2019; Day and Higgins 2015; Huang et al. 2022), indicating a need for more consistent practices, including further education of healthcare staff about delirium, its definition, risk factors and management plans. Drawing on lessons from ICU care, case conferences and family centred rounds, where family members are active participants in the medical rounds, provide families with structured opportunities to voice concerns, seek clarification and participate in care planning, which has been shown to reduce feelings of uncertainty and improve communication between families and clinicians (Davidson et al. 2017). Translating this approach to acute care settings where delirium occurs could ensure that families receive timely and consistent support tailored to the unique challenges of delirium.

Family members frequently reported feeling overwhelmed, anxious and helpless when confronted with the sudden onset of delirium in their loved ones (Assa et al. 2021; Day and Higgins 2015). These emotions seem to cut across all care settings, including acute care, palliative care and delirium‐specific units (Finucane et al. 2017; Toye et al. 2012). Another prevalent emotion experienced by family members that is worth noting is distress, with up to 66% reporting experiencing severe distress related to their family members delirium (Martins et al. 2018). This feeling of being distressed is often linked to the uncertainty surrounding the cause of the delirium, its management and its potential impact on the patient's health outcomes (Assa et al. 2021). The sudden and often unpredictable nature of delirium, and the emotional toll of witnessing cognitive and behavioural changes in their loved ones contribute significantly to the distressed feelings (Day and Higgins 2015; Huang et al. 2022). The emotional toll of witnessing a loved one in a state of confusion due to delirium can also lead to feelings of depression and heightened anxiety among family members. These feelings of depression can present as persistent sadness, feelings of hopelessness, loss of interest, fatigue and negative self‐perception that can affect daily functioning and well‐being (American Psychiatric Association 2013), highlighting the emotional impact delirium has on family members which can be long lasting. Anxiety rates among family members dealing with delirium can range from 22% to 35%, while depression rates vary from up to 54.9% (Poulin et al. 2021; Rosgen et al. 2021). Engaging families in the care process may help reduce their anxiety and foster coping mechanisms. Despite the significant emotional burden described, there remains a notable gap in the provision of sufficient support services to assist families in coping with these challenges. the implementation of delirium specific follow‐up clinics could represent an essential component of comprehensive support. Post‐ICU follow‐up clinics, which have been shown to mitigate long‐term psychological impacts on ICU survivors and their families, could serve as a model (Schofield‐Robinson et al. 2018). A delirium‐specific clinic could provide families with information about delirium, counselling services and opportunities to discuss unresolved questions or concerns with healthcare providers. Such services could address lingering distress and help families understand their loved one's cognitive recovery trajectory, fostering resilience and preparedness for future care needs. Access to social workers and psychologists within acute care settings is another key element of comprehensive support (Davidson et al. 2017). These professionals could provide immediate emotional support, grief counselling and guidance on navigating the complexities of delirium care, as recommended in guidelines for FCC (Davidson et al. 2017). Family‐centred education programmes tailored to delirium are also essential and could include information on recognising delirium symptoms, managing patient behaviours and coping strategies for families (Lange et al. 2022b).

Despite these challenges, families express a strong desire to be involved in their loved one's care (Day and Higgins 2015). Many feel excluded from the care process, which exacerbates their distress and contributes to feelings of helplessness (Naaktgeboren et al. 2022). Involving families in care planning, decision‐making and patient support can enhance their confidence and ability to contribute meaningfully to their loved one's recovery. Structured interventions, such as delirium‐specific education programmes and inclusion in multidisciplinary rounds, can provide families with the knowledge and resources they need to actively participate in care (Bull et al. 2016; Lange et al. 2022b). The Patient and Family‐Centred Care (PFCC) framework provides a structured approach to engaging families, incorporating principles of respect, information sharing, participation and collaboration (Johnson and Abraham 2012). Implementing PFCC in acute care settings can help address families' concerns, improve communication and foster meaningful family involvement in delirium care.

Mapping the existing literature in this scoping review underscores the need for targeted interventions to enhance families' understanding of delirium, improve healthcare communication practices, provide emotional support and integrate families into patient care. Future research should explore the optimal timing and methods for delivering family education, as well as strategies to ensure families receive consistent and comprehensive support throughout their loved one's care journey.

## 7 Limitations

This review has several limitations, including a paucity of primary studies on delirium in the acute care setting from the family's perspective solely; therefore, studies that included patients and staff members perspectives had to be included, which restricted the depth of insight into the family members own experiences by diluting the results. Additional limitations identified included inadequate participant and study design descriptions, small sample sizes and reliance on convenience sampling, which reduces the generalisability of findings. Furthermore, a lack of control for confounding factors, such as variations in patient illness severity, may affect the generalisability of the findings. A limitation of this study was that the included studies did not explicitly include the social situations of families in the analysis or the ages of family members, both of which may limit the generalisability of the findings.

## 8 Conclusion

This scoping review has highlighted the profound impact of delirium on family members of patients in acute care settings. Family members often face intense distress, anxiety and feelings of helplessness due to sudden behavioural and cognitive changes in their loved ones. The lack of a clear understanding and support further intensifies these challenges, underscoring the need for strategies that address their emotional, psychological and practical needs.

Effective communication between healthcare providers and family members is essential to reduce distress and improve family involvement in patient care. Yet, gaps in communication persist, often hindering family members' understanding and participation. Educational initiatives are also critical, as they empower families to actively support their loved ones through the delirium care journey.

This review emphasises actionable improvements for clinical practice, including the adoption of FCC, the implementation of targeted education for high‐risk patients and their families, and improved staff training to enhance insights into delirium from a family perspective.

In conclusion, addressing the diverse needs of family members requires a holistic approach that integrates clear communication, education and support services. By implementing these strategies in clinical settings and prioritising further research in these areas, healthcare systems can improve the experience and well‐being of families dealing with delirium in acute care.

## Author Contributions

All authors made substantial contributions to conception and design, or acquisition of data, or analysis and interpretation of data: involved in drafting the manuscript or revising it critically for important intellectual content; given final approval of the version to be published; agreed to be accountable for all aspects of the work in ensuring that questions related to the accuracy or integrity of any part of the work are appropriately investigated and resolved. Each author should have participated sufficiently in the work to take public responsibility for appropriate portions of the content.

## Conflicts of Interest

The authors declare no conflicts of interest.

## Supporting information


**Appendix S1.** Search terms.

## Data Availability

Data sharing not applicable—no new data generated from this scoping review.
