# Supplementary material for: Delirium in the Acute Care Setting From the Families Perspective: A Scoping Review
Source: J Adv Nurs. 2025 Mar 30;82(1):44–60. doi: 10.1111/jan.16891 (PMC12721945; doi:10.1111/jan.16891)
Supplement: Supplementary file 1 — Appendix S1. Search terms. [file JAN-82-44-s001.docx]

## Appendix A – Search terms

CINHAL: <http://ezproxy.ecu.edu.au/login?url=https://search.ebscohost.com/login.aspx?direct=true&db=cul&bquery=((family)+OR+(relatives+OR+family+OR+partner+OR+loved+one+OR+next+of+kin+OR+spouse+OR+significant+others)+OR+(carers+OR+caregivers+OR+family+members+OR+relatives+OR+informal+carers))+AND+((delirium+OR+acute+confusion+OR+confusion+OR+disorientation)+OR+(delirium+prevention+OR+preventing+delirium+OR+prevent+delirium+OR+reduce+delirium))+AND+((acute+care+setting+OR+hospital)+OR+(post+operative+OR+post-operative+OR+postoperative+OR+post+surgery)+OR+(intensive+care+unit+OR+icu+OR+critical+care+OR+critical+care+unit))+AND+((family+attitudes+OR+experiences)+OR+(perspectives+OR+views+OR+perceptions+OR+attitudes+OR+opinion+OR+understanding+OR+experience))&type=1&searchMode=Standard&site=ehost-live&scope=site>

MEDLINE: <http://ezproxy.ecu.edu.au/login?url=https://search.ebscohost.com/login.aspx?direct=true&db=cmedm&bquery=((carers+OR+caregivers+OR+family+members+OR+relatives+OR+informal+carers)+OR+(relatives+OR+family+OR+partner+OR+loved+one+OR+next+%26quot%3bof%26quot%3b+kin+OR+spouse+OR+significant+others))+AND+((delirium+OR+acute+confusion+OR+confusion+OR+disorientation)+OR+(delirium+prevention+OR+preventing+delirium+OR+prevent+delirium+OR+reduce+delirium))+AND+((acute+care+setting+OR+hospital)+OR+(post+operative+OR+post-operative+OR+postoperative+OR+post+surgery)+OR+(intensive+care+unit+OR+icu+OR+critical+care+OR+critical+care+unit))+AND+((family+attitudes+OR+experiences)+OR+(perspectives+OR+views+OR+perceptions+OR+attitudes+OR+opinion+OR+understanding+OR+experience))&type=1&searchMode=Standard&site=ehost-live&scope=site>
